# Bruxism Control in a Child with Cerebral Palsy

**DOI:** 10.5402/2011/146915

**Published:** 2010-12-01

**Authors:** Cristiana Aroeira G. R. Oliveira, Viviane Andrade Cancio de Paula, Maristela Barbosa Portela, Laura Salignac Guimarães Primo, Gloria Fernanda Castro

**Affiliations:** ^1^Department of Paediatric Dentistry and Orthodontics, School of Dentistry, Federal University of Rio de Janeiro, Rio de Janeiro, 21941-901/RJ, Brazil; ^2^Department of Clinics and Pediatric Dentistry, Fluminense Federal University (UFF), Niterói, Brazil; ^3^Caixa Postal 68066 Cidade Universitária (CCS), Rio de Janeiro RJ, CEP: 21941-971, Brazil

## Abstract

Cerebral palsy (CP) is one of the most severe childhood disabilities due to a lesion in the developing brain. Oral conditions often observed in this pathogenic are a tendency for the delayed eruption of permanent molars, higher percentages of malocclusion and parafunctional habits, including bruxism. The significance of oral conditions observed in CP patients demonstrates the need for intensive home and professional care for these individuals. This paper presents a 7-year-old boy, with cerebral palsy, severe mental retardation, who had high abrasion wear of the primary teeth related to bruxism. Dental care was carried out under oxide-induced sedation, and management of the bruxism was achieved after the use of a resin acrylic protective appliance fixed on both sides of the mandibula. The treatment performed offered efficiency advantages, was clinically viable, and should be a valuable option to practitioners considering appliance therapy to control parafunctional behavior.

## 1. Introduction

Cerebral palsy (CP) is a severe childhood disability, characterized by a nonprogressive motor disorder of posture and movement due to a lesion in the developing brain [1]. The prevalence of this clinical condition among children aged 3–10 years has been reported to be 2.4 in every 1000 live-borns [2], being significantly higher in males and black people. The most common disorders associated with CP are mental retardation, sensory limitations, epilepsy, speech disorders and hearing loss [3]. The more common oral conditions in individuals with CP include higher mean decayed, missing and filled surfaces index, higher plaque index, tendency for delayed eruption of permanent molars, malocclusion [4], as well as high rates of bruxism [5]. 

Bruxism means grinding or gnashing of the teeth [6]. This rhythmic grinding can cause masseter hypertrophy, headaches, temporomandibular joint destruction, and tooth wear. The incidence of bruxism in the general population has been reported to be as high as 21%, but its incidence in PC is still unknown [7]. Many factors may be involved in the etiology of this parafunctional activity such as spasticity [8]; unbalanced oral myofunctional disturbance[3, 5], backbone dysfunction with the head projected forward, which changes the contact between the teeth and predisposes hyperactivity of the main masticatory muscles (temporal and masseteric) [9], lack of control of the mandibular posture which can worsen in periods of emotional stress [10]; sleep disorders [11]; use of neuroleptics [5] and malocclusion [12]. Some of these changes are very common in children with PC [5].

The mastigatory musculature spasticity of CP can interfere with daily activities such as tooth brushing, cleaning of the oral cavity and eating [7]. The treatment for this parafunctional activity includes restorative treatment, occlusal adjustment[9], the use of oral splints, pharmacological treatments and dental extraction [6]. The most severe cases require a multidisciplinary input, including pediatricians, psychiatrists, paediatric dentists, and/or oral surgeons [13].

 The following paper reports a severe case of bruxism in a child with cerebral palsy and discusses the treatment given.

## 2. Case Report

A seven-year-old boy with spastic cerebral palsy (type quadriplegia, the severest CP) was brought to the Pediatric Dentistry Department of the Federal University of Rio de Janeiro, Brazil because his parents complained of the repeated grinding which was damaging his teeth and gums. The parents reported during anamnesis that the pregnancy and parturition were normal and he was born in 40th week of gestation. When he was 5 days old he spent 11 days in the neonatal intensive care unit because of icterus with moderate bilirubin levels and he needed a blood transfusion. 

 The child was under medical treatment during his first year of life because of convulsive crises. He has always been cared for by his parents for all daily actives. The child has been followed up by a multidisciplinary team including pediatrician, physiotherapist, psychologist and neurologist. Medicines taken by the patient daily included Gardenal, Lorenal, Motile and Rivertril.

Clinically, there were no extraoral findings of note. Intraoral examination revealed a mixed dentition and good oral hygiene. All deciduous teeth presented severe dental wear, indicating a pronounced bruxism. The enamel had been worn away on large areas of anterior cusps and mastigatory primary molar surfaces. Because of the poor participation of the patient, the size of movements could not be measured. He had no salivary drooling and had an adequate swallow in spite of evidence of facial and hypoglossal nerve dysfunction. His parents reported he used to eat ordinary food, but in the last six months they had noticed he appeared in pain while eating so he was only able to eat puréed food. 

**Figure 1:**
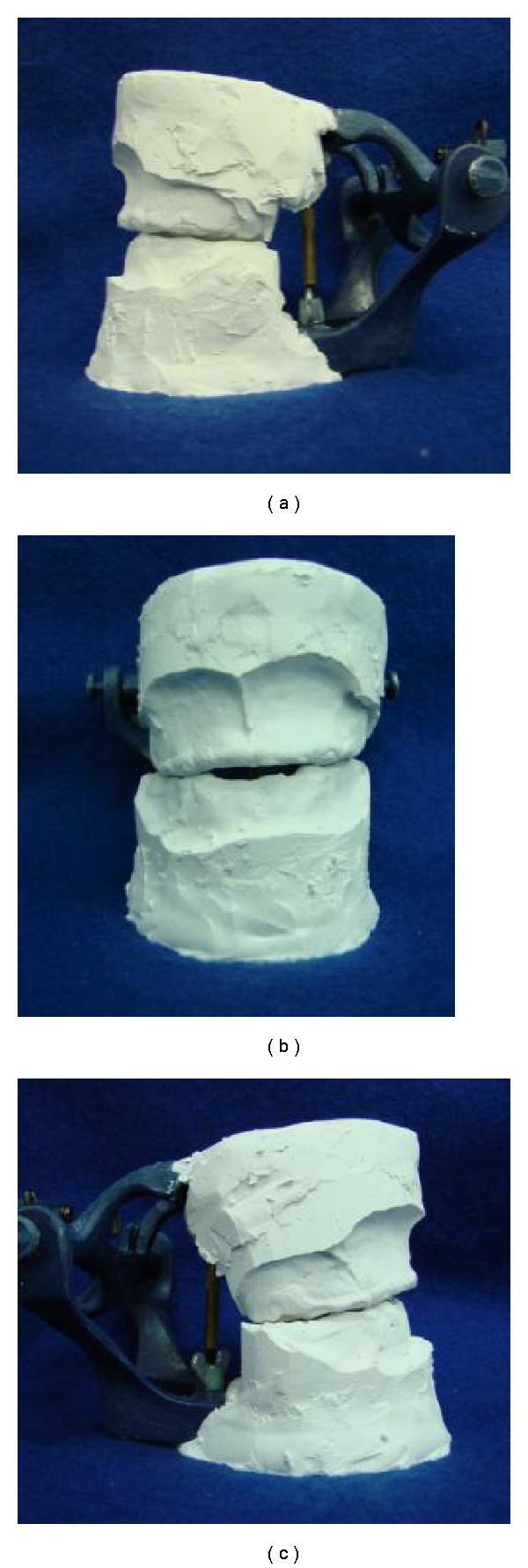
Inicial models. Lateral and frontal vision. The high abrasive wear of primary teeth and the delayed eruption of permanent incisors can be seen.

**Figure 2:**
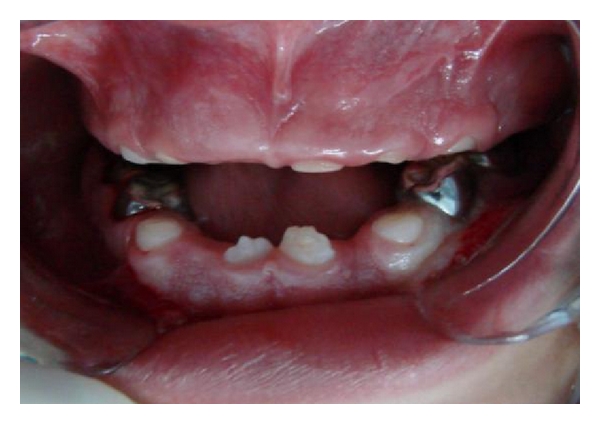
Frontal vision after the adaptation of stainless steel crowns, showing the eruption of the permanent incisors.

**Figure 3:**
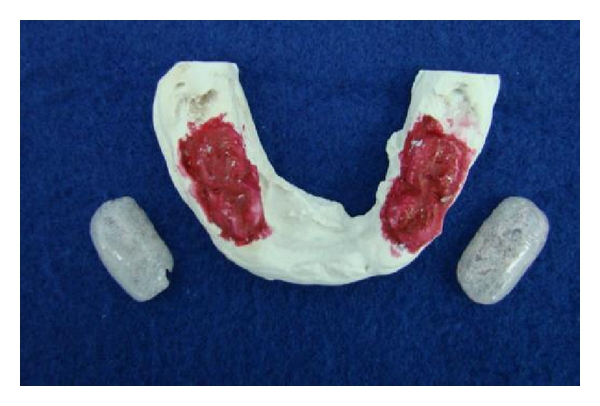
Models used to make the oral appliances before being cemented on the patients primary molars.

**Figure 4:**
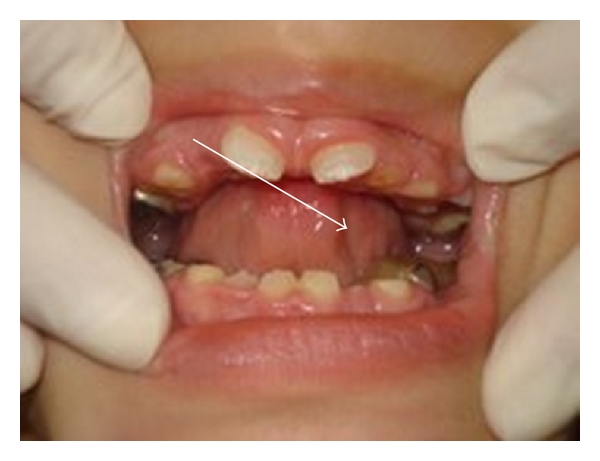
One-year-review: the crowns of permanent central incisors can be seen.

 As the molars had extensive teeth surface losses, stainless steel crowns were the proposed treatment for the primary molars under local anesthesia and nitrous oxide-induced sedation (N_2_O) [14]. At the first appointment, the treatment of the right molars was carried out and four stainless steel crowns were cemented on the primary molars. Seven days later the same treatment was performed on the left primary molars. A week later a protective oral appliance was designed for covering all the maxillary molars, in order to reduce the bruxism and prevent injuries to the soft tissues [15]. Impressions were made with a silicone material to obtain a working model (Figure 1) and two acrylic resin appliances were made (Figures 2 and 3) under N_2_O sedation. At the following appointment, the protective appliance was fixed on both sides of the mandibular teeth by means of ionomer cement. The parents were educated on the proper oral hygiene measures to be adopted and the need for regular dental visits in the future. After a period of two weeks, grinding behavior had decreased significantly and no further damage to the dentition were seen. His parents reported that the child had received the appliance well and he no longer ground his teeth (the bruxism had been controlled with concomitant alleviation of symptoms); feeding had improved, because he was able to consume solid foods instead of the semisolid diet that he had before treatment. The patient was periodically followed up in order to remove and clean the appliances, apply topical fluoride and instruct the parents on oral hygiene. Six months later, the eruption of permanent central incisors of both jaws could be noted and at the one year (Figure 4) review the crowns of these teeth were more exposed. The child remains under continued intervention through a multidisciplinary team.

## 3. Discussion

Individuals with CP tend to develop accentuated involuntary muscle tonus in orofacial muscles and other muscles and often show various types of stereotypy [16], especially when they lack any other occupation, as in this case, where the child occupied himself by grinding his teeth for a large part of the day. Lindqvist and Heijbel [16] observed that abnormal dental wear is closely related to a low level of mental development [17] and severe dental wear indicates that CP children have more pronounced bruxism than normal children [8, 9].

 In this case, clinical signs as lip biting or small ulcers were not found, even though they have been reported as clinical signs of bruxism [6, 18].On the other hand, the patient presented limited mouth opening or trismus, an inherent characteristic of bruxism [18]. 

There is contradictory information in the literature regarding the incidence of oral diseases in patients with CP. According to Brown and Schodel [17], these controversies are due to the failure of the criteria used to choose the population to be studied as well as the absence of control groups. Santos [4] observed a high-risk for dental caries in CP children. However, no carious lesions were diagnosed in the patient described in the present case. This may be explained because the parents had received oral hygiene and diet instructions in a pediatric clinic when the child was very young and they have always been very careful with the child's oral hygiene.

Dental treatment for handicapped patients presents multiple difficulties. They are often treated under general anesthetic or deep sedation, which has many disadvantages [19].Yoshida et al. [14] presented a study pointing out the beneficial properties of nitrous oxide-(N_2_O-) induced sedation performed during dental treatment on CP patients. The authors decided to carry out the dental procedures on the patient using N_2_O sedation considering the procedure was of short duration, the difficulties involved in local hospital admission and the safety of the technique [20].

In studies of bruxism in mentally retarded children in the literature, the term “abnormal abrasion” has varying implications; so treatment for the oral injuries caused by this parafunctional activity may include restorative techniques, dental extractions [21], and the use of oral protective appliances [22]. The first choice was to restore the primary molars with stainless steel crowns because of their high degree of dental wear. This procedure was not sufficiently effective, since the patient continued to grind his teeth with great force. Therefore it was decided to make fixed acrylic appliances to reduce the bruxism. Although the appliance was cemented, the parents were instructed to verify the stability of the appliance in order to avoid the risk of aspiration. Although the appliance was cemented, before choosing this treatment option, the authors discussed the risk of aspiration with the child's parents. They were instructed to verify the stability of the appliance and to keep it cleaned. Although there are few studies concerning the use of oral devices directly with children, an oral appliance applied to prevent the self-mutilation of the lower lip in a mentally handicapped patient with Moebius syndrome, has been described, with positive results [15, 23]. The bruxism was reduced and the parents related that activities like eating and sucking became easier for the patient. It probably occurred because the resin appliance increased vertical dimension and the occlusal parameters had been established, so the patient was able to have a better functional occlusal activity.

 In a later followup, the eruption of permanent first molars and incisors was observed, indicating the success of the treatment. As the child gets older, the appliance can be extended to other teeth if necessary, as his future neurological condition cannot be predicted.

Early diagnosis and interventions of parafunctional habits in individuals with CP is important to reduce invasive treatment and worse complications for the patient. This paper also highlights that oral protective appliances can be clinically viable and effective for the prevention of bruxism. Furthermore, this case illustrates the importance of the treatment by a dental team in patients with cerebral palsy.
